# Association Between CMS Quality Ratings and COVID-19 Outbreaks in Nursing Homes — West Virginia, March 17–June 11, 2020

**DOI:** 10.15585/mmwr.mm6937a5

**Published:** 2020-09-18

**Authors:** David P. Bui, Isaac See, Elisabeth M. Hesse, Kate Varela, R. Reid Harvey, Euna M. August, Andrea Winquist, Samantha Mullins, Shannon McBee, Erica Thomasson, Amy Atkins

**Affiliations:** ^1^Epidemic Intelligence Service, CDC; ^2^Division of Environmental Health Science and Practice, National Center for Environmental Health, CDC; ^3^Division of Healthcare Quality Promotion, National Center for Emerging and Zoonotic Infectious Diseases, CDC; ^4^Division of Preparedness and Emerging Infections, National Center for Emerging and Zoonotic Infectious Diseases, CDC; ^5^National Institute for Occupational Safety and Health, CDC; ^6^Division of HIV/AIDS Prevention, National Center for HIV/AIDS, Viral Hepatitis, STD, and TB Prevention, CDC; ^7^West Virginia Bureau for Public Health, West Virginia Department of Health and Human Resources; ^8^Division of State and Local Readiness, Center for Preparedness and Response, CDC.

Nursing homes are high-risk settings for outbreaks of SARS-CoV-2, the virus that causes coronavirus disease 2019 (COVID-19) (*1*,*2*). During the COVID-19 pandemic, U.S. health departments worked to improve infection prevention and control (IPC) practices in nursing homes to prevent outbreaks and limit the spread of COVID-19 in affected facilities; however, limited resources have hampered health departments’ ability to rapidly provide IPC support to all nursing homes within their jurisdictions. Since 2008, the Centers for Medicare & Medicaid Services (CMS) has published health inspection results and quality ratings based on their Five-Star Quality Rating System for all CMS-certified nursing homes (*3*); these ratings might be associated with facility-level risk factors for COVID-19 outbreaks. On April 17, 2020, West Virginia became the first state to mandate and conduct COVID-19 testing for all nursing home residents and staff members to identify and reduce transmission of SARS-CoV-2 in these settings (*4*). West Virginia’s census of nursing home outbreaks was used to examine associations between CMS star ratings and COVID-19 outbreaks. Outbreaks, defined as two or more cases within 14 days (with at least one resident case), were identified in 14 (11%) of 123 nursing homes. Compared with 1-star–rated (lowest rated) nursing homes, the odds of a COVID-19 outbreak were 87% lower among 2- to 3-star–rated facilities (adjusted odds ratio [aOR] = 0.13, 95% confidence interval [CI] = 0.03–0.54) and 94% lower among 4- to 5-star–rated facilities (aOR = 0.06, 95% CI = 0.006–0.39). Health departments could use star ratings to help identify priority nursing homes in their jurisdictions to inform the allocation of IPC resources. Efforts to mitigate outbreaks in high-risk nursing homes are necessary to reduce overall COVID-19 mortality and associated disparities. Moreover, such efforts should incorporate activities to improve the overall quality of life and care of nursing home residents and staff members and address the social and health inequities that have been recognized as a prominent feature of the COVID-19 pandemic in the United States (*5*).

COVID-19 surveillance data from the West Virginia Department of Health and Human Resources were used to identify all nursing home outbreaks during March 14–June 11, 2020. These outbreaks were identified through routine COVID-19 surveillance and by universal nursing home testing, which was conducted per the governor’s executive order* during April 21–May 8, 2020 (*4*). For this report, an outbreak was defined as two or more laboratory-confirmed SARS-CoV-2 cases occurring within 14 days in a nursing home, with at least one of those cases in a resident.

Nursing home data were downloaded from the CMS Nursing Home Compare website^†^ on June 11, 2020, and included data on all CMS-certified nursing homes (*3*). CMS-trained inspectors conduct annual unannounced health inspections of all nursing homes; inspection deficiencies are recorded, scored, and summarized into an overall five-star rating (1 star = lowest quality, 5 star = highest quality) that is adjusted based on nursing home staffing levels (e.g., nursing hours per resident) and quality of care measures (e.g., hospital readmissions). This analysis is based on star ratings from the most recent nursing home inspections in West Virginia, conducted during December 13, 2018–February 26, 2020, approximately 2 weeks before the first reported COVID-19 case in the state. Most inspections were conducted in 2019 (101 of 123; 82%) and 2020 (21; 17%); one inspection was conducted in 2018.

Wilcoxon rank-sum tests were used to evaluate continuous variables and Fisher’s exact tests for categorical variables, to compare facilities with and without COVID-19 outbreaks (outbreak- and nonoutbreak facilities) on several CMS survey measures, including ownership type, average daily number of residents, average daily staffing hours per resident, cumulative county-level COVID-19 incidence, and number of CMS inspection deficiencies, fines, and penalties. P-values <0.05 were considered statistically significant. Logistic regression models were used to assess the association between overall star ratings and COVID-19 outbreaks, adjusting for county-level COVID-19 incidence (analyzed as continuous cases per 100,000 population) and average daily number of facility residents (analyzed as continuous number of facility residents per day). To facilitate interpretation of the OR for county-level incidence and average daily number of facility residents, the variables were rescaled by a factor of 10 (i.e., divided by 10). The overall star rating was analyzed as a three-level variable (1-star, 2–3-star, and 4–5-star). The outcome of interest was experiencing a COVID-19 outbreak, and the reference group was 1-star–rated nursing homes. ORs and 95% CIs were estimated with R statistical software (version 3.6.1; The R Foundation). This activity was reviewed by CDC and was conducted consistent with applicable federal law and CDC policy.^§^

As of June 11, 2020, there were 123 CMS-certified nursing homes in West Virginia, including 18 (15%) rated as 5-star, 22 (18%) as 4-star, 28 (23%) as 3-star, 34 (28%) as 2-star, and 20 (16%) as 1-star; one (1%) nursing home was unrated (Table 1). Most (19 of 20, 95%) 1-star–rated nursing homes were for-profit operations and tended to have more residents than did higher rated nursing homes. Nurse staffing levels were generally lower in 1-star–rated facilities, compared with those in higher rated facilities (Table 1).

**TABLE 1:** Nursing home characteristics, staffing levels, and county characteristics of all Centers for Medicare & Medicaid Services–certified nursing homes, by overall star rating — West Virginia, 2020

Characteristic	Overall star rating, mean (95% CI)					
	1 Star n = 20	2 Star n = 34	3 Star n = 28	4 Star n = 22	5 Star n = 18	All* n = 123
For-profit nursing home, no. (%)	19 (95.0)	27 (79.4)	21 (75.0)	17 (77.3)	10 (55.6)	95 (77.2)
No. of certified beds	107 (88–126)	94 (80–107)	82 (71–93)	83 (65–101)	61 (40–81)	87 (80–93)
No. of facility residents per day	95 (78–111)	85 (73–97)^†^	75 (64–85)	70 (57–82)	56 (36–76)	77 (71–84)
**Nurse staffing level**						
Nurse aide hours per resident per day	2.0 (1.8–2.2)^†^	2.2 (2.1–2.4)^†^	2.1 (2.0–2.3)	2.2 (2.0–2.4)	2.4 (2.2–2.6)	2.2 (2.1–2.3)
Registered nurse hours per resident per day	0.5 (0.4–0.6)^†^	0.6 (0.5–0.7)^†^	0.6 (0.5–0.7)	0.6 (0.5–0.7)	1.2 (0.7–1.7)	0.7 (0.6–0.8)
Total nurse hours per resident per day	3.4 (3.2–3.6)^†^	3.7 (3.5–3.9)^†^	3.7 (3.4–3.9)	3.7 (3.5–4)	4.7 (3.9–5.5)	3.8 (3.6–4)
**Facility county characteristic**						
County population (x10,000)	9.6 (6.5–12.7)	5.5 (4.0–7.1)	4.1 (2.7–5.5)	4.4 (3.2–5.7)	5.0 (2.2–7.9)	5.6 (4.7–6.5)
County-level COVID–19 incidence^§^	113 (68–159)	109 (74–144)	143 (84–203)	101 (65–138)	92 (60–124)	113 (94–132)

**Abbreviations:** CI = confidence interval; COVID-19 = coronavirus disease 2019.

* One nursing home did not receive a star rating.

^†^ One nursing home not reporting.

^§^ County level COVID-19 cases per 100,000 population; calculated based on cumulative county case counts as of June 11, 2020.

As of June 11, the West Virginia Department of Health and Human Resources reported COVID-19 outbreaks in 14 (11%) nursing homes, with 226 cases among residents (median = 2.5 per nursing home, range = 1–71) and 140 cases among staff members (median = 4, range = 0–39). Average daily resident census in outbreak facilities (92) was higher than that in nonoutbreak facilities (76) (p = 0.03) (Table 2). Total nurse staffing hours per resident per day were similar in outbreak and nonoutbreak facilities, but mean number of nurse aide hours per resident per day in outbreak facilities (1.9) was lower than was that in nonoutbreak facilities (2.2) (p = 0.02). COVID-19 incidence was higher in counties where outbreak facilities were located (mean = 178 per 100,000) compared with that in counties where nonoutbreak facilities were located (105 per 100,000) (p = 0.001). The mean number of health deficiencies was higher in outbreak facilities (mean = 15) than in nonoutbreak facilities (mean = 11) (p = 0.03) (Table 3).

**TABLE 2:** Nursing home characteristics, staffing levels, and county characteristics, by COVID-19 outbreak status — West Virginia, March 17–June 11, 2020

Characteristic	Nursing home outbreak* status, mean (95% CI)			P-value^†^
	Nonoutbreak n = 109	Outbreak n = 14	All n = 123	
For-profit nursing home, no. (%)	82 (75.2)	13 (92.9)	95 (77.2)	0.19
No. of certified beds	84.6 (77.0–92.1)	104.1 (86.0–122.2)	86.8 (79.8–93.8)	0.05
No. of facility residents per day	75.6 (68.9–82.4)^§^	92.2 (79.6–104.8)	77.5 (71.3–83.7)	0.03
**Nurse staffing level**				
Nurse aide hours per resident per day	2.2 (2.1–2.3)^¶^	1.9 (1.7–2.1)	2.2 (2.1–2.3)	0.02
Registered nurse hours per resident per day	0.7 (0.6–0.8)^¶^	0.6 (0.5–0.7)	0.7 (0.6–0.8)	0.90
Total nurse staffing hours per resident per day	3.8 (3.7–4.0)^¶^	3.5 (3.2–3.8)	3.8 (3.6–4.0)	0.22
**Facility county characteristic**				
County population (x10,000)	5.1 (4.3–5.9)	9.3 (5.0–13.7)	5.6 (4.7–6.5)	0.08
County-level incidence**	105.1 (85.6–124.6)	177.8 (108.4–247.2)	113.4 (94.3–132.5)	0.001

**Abbreviations:** CI = confidence interval; COVID-19 = coronavirus disease 2019.

* An outbreak was defined as two or more confirmed cases detected in a nursing home within 14 days, with at least one case in a resident.

^†^ P-values based on Wilcoxon rank-sum test (for continuous variables) and Fisher’s exact test (for categorical variables).

^§^ One nursing home not reporting.

^¶^ Two nursing homes not reporting.

** County level COVID-19 cases per 100,000 population; calculated based on cumulative county case counts as of June 11, 2020.

**TABLE 3:** Summary of overall star rating* and health inspection deficiencies† of nursing homes — West Virginia, 2020

Characteristic	Outbreak status			P-value^§^
	Nonoutbreak n = 109	Outbreak n = 14*	All n = 123	
**Overall star rating, no. (%)**				
1 Star	13 (12)	7 (50)	20 (16)	<0.001
2 Star	34 (31)	0 (0)	34 (28)	
3 Star	23 (21)	5 (36)	28 (23)	
4 Star	21 (19)	1 (7)	22 (18)	
5 Star	18 (17)	0 (0)	18 (14)	
**Deficient infection prevention control program, no. (%)^†,¶^**				
Within last year	69 (63)	12 (86)	81 (66)	0.14
Within last 2 years	90 (83)	14 (100)	104 (85)	0.12
**Summary of complaints, fines, and deficiencies, mean (95% CI)^†^**				
No. of substantiated complaints**	1.3 (0.8–1.8)	4.8 (1.6–8.0)	1.7 (1.1–2.3)	<0.001
No. of health inspection deficiencies	10.5 (9.2–11.9)	14.9 (10.5–19.2)	11.0 (9.7–12.3)	0.03
No. of penalties	0.2 (0.1–0.4)	0.5 (0.1–0.9)	0.3 (0.2–0.4)	0.06
No. of fines	0.2 (0.1–0.3)	0.4 (0.1–0.8)	0.2 (0.2–0.3)	0.17
**Counts of health inspection deficiencies by category, mean (95% CI)^†^**				
Quality of life and care	2.4 (2.0–2.8)	3.8 (2.6–5.0)	2.6 (2.2–2.9)	0.01
Resident assessment and care planning	2.2 (1.9–2.5)	3.5 (2.9–4.1)	2.3 (2.1–2.6)	<0.001
Nursing and physician services	0.4 (0.3–0.5)	0.6 (0.2–0.9)	0.4 (0.3–0.5)	0.15
Resident rights	1.9 (1.6–2.3)	1.8 (0.9–2.7)	1.9 (1.6–2.2)	0.89
Nutrition and dietary	0.8 (0.6–1.0)	1.4 (0.4–2.3)	0.9 (0.7–1.1)	0.24
Pharmacy service	1.0 (0.8–1.2)	1.2 (0.8–1.7)	1.0 (0.8–1.2)	0.21
Environmental	1.0 (0.8–1.1)	1.2 (0.8–1.7)	1.0 (0.9–1.1)	0.35
Administration	0.4 (0.2–0.5)	0.8 (0–1.6)	0.4 (0.3–0.6)	0.26

**Abbreviation:** CI = confidence interval.

* Only 13 outbreak facilities received a star rating; one outbreak nursing home was designated a Special Focus Facility and not rated because of a history of serious quality issues.

^†^ These health inspection deficiencies were recorded during unannounced inspections conducted during December 13, 2018–February 26, 2020.

^§^ P-values based on Wilcoxon rank-sum test (for continuous variables) and Fisher’s exact test (for categorical variables).

^¶^ This CMS inspection finding based on the requirement that “the facility must establish and maintain an infection prevention and control program designed to provide a safe, sanitary, and comfortable environment and to help prevent the development and transmission of communicable diseases and infections.” Refer to 42 C.F.R. Sect. 483.80 for full requirements.

** Number of concerns or complaints (related to abuse, neglect, poor care, insufficient staffing, unsafe or unsanitary conditions, dietary problems, or mistreatment) reported to CMS that were investigated and substantiated; inspectors responsible for annual health inspections are federally required to investigate all complaints.

Seven (50%) of 14 outbreak facilities had 1-star ratings compared with 13 (12%) of 109 nonoutbreak facilities (Table 3). One outbreak facility was a CMS-designated Special Focus Facility and did not receive a star rating and was not included in regression analysis. Special Focus Facility designation is reserved for the lowest rated facilities in the state with a history of serious inspection deficiencies (i.e., potential to harm residents). In unadjusted analyses, the odds of a COVID-19 outbreak in a nursing home increased by 5% for each additional 10 incident cases per 100,000 in the county (OR = 1.05, 95% CI = 1.00–1.09) and by 14% for each additional 10 facility residents (OR = 1.14; 95% CI = 0.98–1.33). Compared with 1-star–rated nursing homes, the unadjusted odds of a COVID-19 outbreak were significantly lower among 2- to 3-star–rated nursing homes (OR = 0.16; 95% CI = 0.04-0.59) and 4- to 5-star–rated nursing homes (OR = 0.05, 95% CI = 0.003). After adjusting for county-level COVID-19 incidence and the number of facility residents, odds of a COVID-19 outbreak were significantly lower in higher quality nursing homes, based on star rating. Compared with 1-star–rated nursing homes, the odds of a COVID-19 outbreak were 87% lower among 2- to 3-star–rated nursing homes (aOR = 0.13; 95% CI = 0.03–0.54) and 94% lower among 4- to 5-star–rated nursing homes (aOR = 0.06; 95% CI = 0.003–0.39); specifically, the odds of a COVID-19 outbreak among 1-star–rated nursing homes were approximately seven times higher than among 2- to 3-star–rated facilities and approximately 17 times higher than among 4- to 5-star–rated facilities after controlling for number of residents and county-level incidence.

## Discussion

West Virginia nursing homes located in counties with high incidences of COVID-19 and those with 1-star ratings have a higher risk of experiencing COVID-19 outbreaks. The odds of a COVID-19 outbreak in 1-star–rated nursing homes were approximately seven times higher than were those in 2- to 3-star–rated facilities and approximately 17 times higher than in 4- to 5-star–rated nursing homes. Early reports have shown that controlling SARS-CoV-2 transmission in nursing homes is challenging (*1*,*2*); however, rapid and early deployment of IPC strategies,^¶^ such as visitor restrictions, use of face masks, staff member education, symptom screening, preparing and implementing outbreak plans, and facility-wide serial testing might successfully prevent or contain outbreaks (*6*). Lower rated nursing homes might struggle to implement effective IPC measures for COVID-19 and might require assistance. Health departments could evaluate the use of CMS star ratings for their facilities to identify priority nursing homes for IPC support and resource allocations to help prevent outbreaks or slow the spread of SARS-CoV-2. Health departments can use resources like the CDC’s COVID-19 Infection Control Assessment and Response** tool to help nursing homes assess outbreak preparedness and implement recommended IPC measures.

Studies have found that nursing homes with low star ratings are associated with a higher risk of health care–associated infections (*7*), worse post-surgery outcomes (*8*), and higher readmission rates following hospitalization (*8*,*9*) compared with those with higher ratings. At least two studies have hypothesized that lower nursing staff levels might underlie the association between low star ratings and resident health outcomes (*8*,*9*). In this report, outbreak facilities had significantly lower nurse aide staffing levels, suggesting that staffing might also be an important factor in outbreak prevention. Low nurse staffing levels might contribute to lower quality of care and could pose challenges to implementing effective IPC strategies including symptom monitoring and rapid detection of COVID-19 in residents. Low nurse staffing levels also might be indicative of under-resourced nursing homes without financial resources to hire sufficient staff or purchase supplies needed for effective IPC, even with health department support.

The findings in this report are subject to at least four limitations. First, CMS star ratings are composite measures of inspection factors, and this study does not identify specific factors driving the association between star rating and outbreak risk; thus, recommendations cannot be made regarding which quality metrics to improve to prevent outbreaks. Therefore, although improving resident care is important, general quality improvement programs without a focus on metrics that strengthen IPC might not lead to reductions in outbreak risk. CMS has responded to the COVID-19 pandemic by guiding the Quality Innovation Network–Quality Improvement Organizations (part of a federal program charged with improving health care quality for Medicare beneficiaries) to low-rated nursing homes, which have a history of IPC challenges and rising incidence and prevalence rates, to address quality issues as well as to provide COVID-19–specific IPC support.^††^ Second, although the models used in these analyses are adjusted for county-level COVID-19 incidence and number of facility residents, there might be additional unaccounted-for confounding factors. For example, data about COVID-19 IPC measures and interventions in place in nursing homes and data on resident demographics were not available yet might be important confounding factors in the apparent association between nursing home quality and outbreak risk. However, confounding might not be a relevant issue if star ratings are used only for risk stratification. Third, the association between star rating and nursing home outbreaks is based on West Virginia’s experience and might not be generalizable to other states or jurisdictions. Finally, staffing and resident estimates provided by CMS were based on annual daily averages and might not reflect actual staffing levels during the analytic period.

Low-rated nursing homes are more likely than are higher rated nursing homes to serve patients experiencing social and economic disadvantage, including dual Medicare-Medicaid enrollees, racial and ethnic minority populations, and persons with low income (*10*) who might already be at higher risk for severe COVID-19 illness and death, thus compounding the risk. The COVID-19 pandemic has highlighted the long-standing inequitable distribution of poor health among many U.S. communities, including among nursing home residents and staff members who shoulder a disproportionate burden of COVID-19 morbidity and mortality (*5*). Efforts to mitigate the risk for outbreaks in high-risk nursing homes are necessary to reduce overall COVID-19 mortality and associated disparities. Moreover, such efforts should incorporate activities to improve the overall quality of life and care of nursing home residents and staff members and address the social and health inequities that have been recognized as a prominent feature of the COVID-19 pandemic in the United States (*5*).

**Summary**

**What is already known about this topic?**

Nursing homes are high-risk settings for COVID-19 outbreaks. The Centers for Medicare & Medicaid Services (CMS) publishes star quality ratings of all CMS-certified nursing homes.

**What is added by this report?**

During March–June 2020, 14 (11%) of 123 West Virginia nursing homes experienced COVID-19 outbreaks. Compared with 1-star–rated (lowest rating) nursing homes, the odds of a COVID-19 outbreak were 87% lower among 2- to 3-star–rated facilities and 94% lower among 4- to 5-star–rated facilities.

**What are the implications for public health practice?**

CMS star ratings can serve as proxy indicators for COVID-19 outbreak risk; health departments could use them to identify priority nursing homes and inform the allocation of infection prevention and control resources.

## Footnotes

* https://governor.wv.gov/Documents/2020%20Executive%20Orders/Executive-Order-April-17-2020-Nursing-Home-Testing.pdf.

† https://data.medicare.gov/data/nursing-home-compare.

§ 45 C.F.R. part 46.102(l)(2), 21 C.F.R. part 56; 42 U.S.C. Sect. 241(d); 5 U.S.C. Sect. 552a; 44 U.S.C. Sect. 3501 et seq.

¶ https://www.cdc.gov/coronavirus/2019-ncov/hcp/long-term-care.html.

** https://www.cdc.gov/coronavirus/2019-ncov/hcp/assessment-tool-for-nursing-homes.html.

†† https://www.cms.gov/files/document/qso-20-31-all.pdf.
